# Association between serum copper levels and prevalence of hyperuricemia: a cross-sectional study

**DOI:** 10.1038/s41598-020-65639-0

**Published:** 2020-05-26

**Authors:** Ting Jiang, Dongxing Xie, Jing Wu, Hongyi He, Haochen Wang, Ning Wang, Zhenglei Zhu, Yilun Wang, Tuo Yang

**Affiliations:** 1 Department of Orthopaedics, Xiangya Hospital, Central South University, Changsha, Hunan China; 2 Department of Ultrasonography, Xiangya Hospital, Central South University, Changsha, Hunan China; 3 Department of Epidemiology and Health Statistics, Xiangya School of Public Health, Central South University, Changsha, Hunan China; 4 Department of Health Management Center, Xiangya Hospital, Central South University, Changsha, Hunan China

**Keywords:** Medical research, Epidemiology

## Abstract

Hyperuricemia has been recognized as a worldwide public health concern. This study was conducted to examine the association between serum copper (Cu) concentration and the prevalence of hyperuricemia in a middle-aged and elderly population. Serum Cu concentration was measured by Roche modular P800 using the PAESA method. Serum uric acid (UA) concentration was detected by a Beckman Coulter AU 5800. Presence of hyperuricemia was defined as serum UA ≥ 416 μmol/L for men and ≥360 μmol/L for women. The association between serum Cu concentration and the prevalence of hyperuricemia was evaluated by logistic regression. The prevalence of hyperuricemia was 17.6% (n = 6,212) in the present study. Relative to the lowest quintile, the age- and sex-adjusted odds ratios for hyperuricemia were 1.38 (95% CI: 1.12 to 1.70), 1.34 (95% CI: 1.07 to 1.66), and 1.53 (95% CI: 1.23 to 1.91) in the third, fourth, and fifth serum Cu concentration quintiles (P for trend < 0.001). Similar results were found both in men and women subgroups. None of the findings were materially altered after adjustment for additional potential confounders. In conclusion, in this population-based cross-sectional study, serum Cu concentration was positively associated with the prevalence of hyperuricemia.

## Introduction

Hyperuricemia is a condition characterized by abnormally elevated levels of serum uric acid (UA). The reported prevalence of hyperuricemia has been showing a rising trend in recent years^1,2^, which ranged from 17.0% to 22.9% in men and 5.9% to 21.6% in women^1,3–6^. Previous studies have suggested that hyperuricemia is associated with hypertension, metabolic syndrome, cardiovascular diseases, chronic kidney diseases, diabetes mellitus, and an increased risk of all-cause mortality^7–12^. However, uncertainties still exist with respect to the specific pathogenesis of hyperuricemia.

Copper (Cu) is one of the essential trace elements involved in many important biological processes of living organisms^13^. Cu *in vivo* has demonstrated both its pro- and anti-oxidant effects^14^. UA is directly oxidized by Cu^2+^^15^, and the Cu-reduction ability of UA was presumed to influence both the pro- and anti-oxidant behaviors of UA^16^. To the best of our knowledge, only one study has attempted to associate serum Cu with serum UA. Bo *et al*.^17^ detected the serum Cu and serum UA concentration in a randomly identified subgroup of men (n = 231) from a study of adults (45- to 64-year-old) in Italy, in which no significant association between serum Cu concentration and UA was concluded. However, the sample size of the study was relatively small and women subjects was not included. Up to now, there has been no research directly related serum Cu to prevalence of hyperuricemia.

To fill in this knowledge gap, we used data collected from a large population-based cross-sectional study to investigate whether there is an association between serum Cu levels and the prevalence of hyperuricemia.

## Materials and methods

### Study population

The present cross-sectional study was performed in Xiangya Hospital, Central South University in Changsha, Hunan Province, China^3,18^. All procedures were conducted in accordance with the relevant guidelines and regulations. In order to acquire data on health-related habits and demographic characteristics, registered nurses performed face-to-face interview on all the subjects during physical examination with a standard questionnaire. The included subjects were chosen based on the criteria as follows: 1) ≥40 years old; 2) receiving serum UA and serum Cu measurements; 3) providing required personal features, including age, sex and body mass index (BMI); 4) providing health-related habits, including activity level, smoking and alcohol drinking status. From October 2013 to December 2015, there were 6212 subjects in all received routine measurements like serum UA and serum Cu, and also provided detailed demographic features and required health-related habits.

### Assessment of hyperuricemia

The blood samples of all the subjects were collected after a 12-hour overnight fast and then preserved at 4 °C until assayed. Serum UA concentration was detected by a Beckman Coulter AU 5800 (Beckman Coulter Inc., Brea, CA, USA), and the results were used to diagnose hyperuricemia (UA ≥ 416 μmol/L for men and ≥360 μmol/L for women).

### Assessment of exposures

The serum Cu concentration was measured by Roche modular P800 using the PAESA method. The inter-assay coefficients of variation were 6.15% (9.8 μmol/L) and 4.325% (16.5 μmol/L), and the intra-assay coefficients of variation were 5.17% (16.72 μmol/L) and 5.37% (8.86 μmol/L) for serum Cu respectively. Fast blood glucose was tested using glucose oxidase enzyme method. Blood pressure was checked on an electronic sphygmomanometer. Diabetes was diagnosed as fasting glucose level ≥7.0 mmol/L or if the subject was receiving drug treatment to control blood glucose. Hypertension was defined as systolic blood pressure ≥140 mmHg or diastolic blood pressure ≥90 mmHg, or if the subject was taking antihypertensive treatment. The BMI of each subject was calculated as weight in kilograms divided by height in meters squared. The information of sports activities, smoking and drinking status was extracted from questionnaires answered by subjects. Sports activities included average frequency and average duration, and current status of smoking and drinking was evaluated in binary (yes or no).

### Statistical analysis

The quantitative data were presented in form of mean ± standard deviation, and the qualitative data were presented in form of percentage. The serum Cu concentration was divided into 5 categories in terms of the quintile distribution of the sample: ≤13.60, 13.61–15.40, 15.41–17.00, 17.01–19.00 and ≥19.01 μmol/L. The one-way classification ANOVA and the Kruskal-Wallis H test was performed to evaluate continuous data for normal distribution and abnormal distribution, and the χ^2^ test was employed to assess the differences between qualitative data. The quintile with the lowest value of serum Cu concentration was regarded as reference, age- and sex- adjusted odds ratios (ORs) and 95% confidence intervals (CIs) were reported to indicate the association between serum Cu and the prevalence of hyperuricemia in each quintile, respectively (Model 1). Moreover, two multivariable models (Model 2 and 3) were also used for logistic regression analyses in the whole, male and female population. The covariate adjusted model 2 included age, sex, BMI, smoking, drinking status, and we additionally adjusted for education, activity level, hypertension, diabetes in subsequent model 3. Median variables of serum Cu concentration in first to the highest quintile were used to test the linear trends by logistic regression. To increase statistical power, we additionally modelled continuous serum Cu concentrations, estimating the effect on hyperuricemia prevalence per 10 μmol/L increase. A scatter plot was employed to test linear relationship between serum Cu and UA after multivariable analyses.

The collected data were conducted by SPSS 17.0 and STATA 12.0; a P value of ≤0.05 was considered as the criterion for statistical significance.

### Ethical approval and informed consent

The study protocol had been approved by the Ethics Committee of Xiangya Hospital, Central South University (reference number: 201312459). Written informed consents had been acquired from all included participants prior to the performance of research.

## Results

A total of 6,212 subjects (3,700 male and 2,512 female) were recruited in the current analysis. The overall prevalence of hyperuricemia in this study was 17.6%. Table 1 shows basic characteristics of the whole population and subjects according to their hyperuricemia status. Significant differences were observed between the hyperuricemia and non-hyperuricemia population in terms of sex, smoking, drinking status, education, BMI, occupation, hypertension and diabetes.

**Table 1:** Basic characteristics of the HU and Non-HU population (n = 6,212).

Basic characteristics	Overall	HU status		*P*
		HU population	Non-HU population	
Number	6,212	1,095	5,117	—
Age (years)	51.93 ± 7.27	51.81 ± 7.28	51.95 ± 7.27	0.409
Sex (% female)	40.4	19.7	44.9	<0.001
Smoking (%)	25.7	30.4	24.7	<0.001
Drinking (%)	41.4	57.4	38.0	<0.001
Education level (% with or above high school background)	47.7	55.4	46.0	<0.001
Activity level (h/week)	2.06 ± 3.26	2.00 ± 3.19	2.07 ± 3.27	0.910
BMI (kg/m^2^)	24.56 ± 3.23	26.07 ± 3.14	24.24 ± 3.15	<0.001
Occupation (% manual worker)	18.1	14.8	18.8	0.002
Hypertension (%)	32.1	47.7	28.7	<0.001
Diabetes (%)	10.1	12.1	9.7	0.024

HU, hyperuricemia; Cu, copper; BMI, body mass index.

Data are mean ± standard deviation, unless otherwise indicated.

*P* values are for the test of difference between the HU population and Non-HU population using one-way analysis of variance in case of normally distributed continuous variables, Kruskal-Wallis H test in case of non-normally distributed continuous variables and Pearson Chi-square test in case of categorical variables.

The results of demographic characteristics and confounding factors are shown in Table 2, in accordance with the quintiles of serum Cu concentration. There were significant differences across all quartiles of serum Cu in terms of age, sex, smoking status, drinking status, education, activity level, BMI, occupation, hypertension and diabetes.

**Table 2:** Basic characteristics of 6,212 participants according to quintiles of serum Cu.

Basic characteristics	Quintiles of serum Cu (μmol/L)					*P*
	Q1 ( ≤ 13.60)	Q2 (13.61–15.40)	Q3 (15.41–17.00)	Q4 (17.01–19.00)	Q5 (≥19.01)	
Number	1,283	1,273	1,233	1,182	1,241	—
Median serum Cu (μmol/L)	12.40	14.60	16.20	17.90	20.90	—
HU (n)	218	232	237	198	210	—
Age (years)	50.38 ± 6.93	51.49 ± 7.20	51.70 ± 7.12	52.41 ± 7.21	53.75 ± 7.47	<0.001
Sex (% female)	23.7	29.5	39.3	50.8	60.2	<0.001
Smoking (%)	32.3	29.3	25.5	21.8	18.9	<0.001
Drinking (%)	46.5	46.2	44.9	36.5	32.4	<0.001
Education level (% with or above high school background)	52.5	52.3	50.3	43.9	38.8	<0.001
Activity level (h/week)	2.03 ± 3.17	2.23 ± 3.32	1.95 ± 3.08	2.03 ± 3.24	2.06 ± 3.45	0.031
BMI (kg/m^2^)	24.81 ± 3.13	24.71 ± 3.17	24.62 ± 3.14	24.45 ± 3.27	24.22 ± 3.39	<0.001
Occupation (% manual worker)	16.9	16.1	18.2	17.5	21.8	0.003
Hypertension (%)	27.8	32.0	32.4	32.7	35.5	0.001
Diabetes (%)	9.0	10.3	9.2	9.8	12.3	0.045

HU, hyperuricemia; Cu, copper; BMI, body mass index.

Data are mean ± standard deviation, unless otherwise indicated.

*P* values are for test of difference across all quintiles of serum Cu.

**Figure 1:**
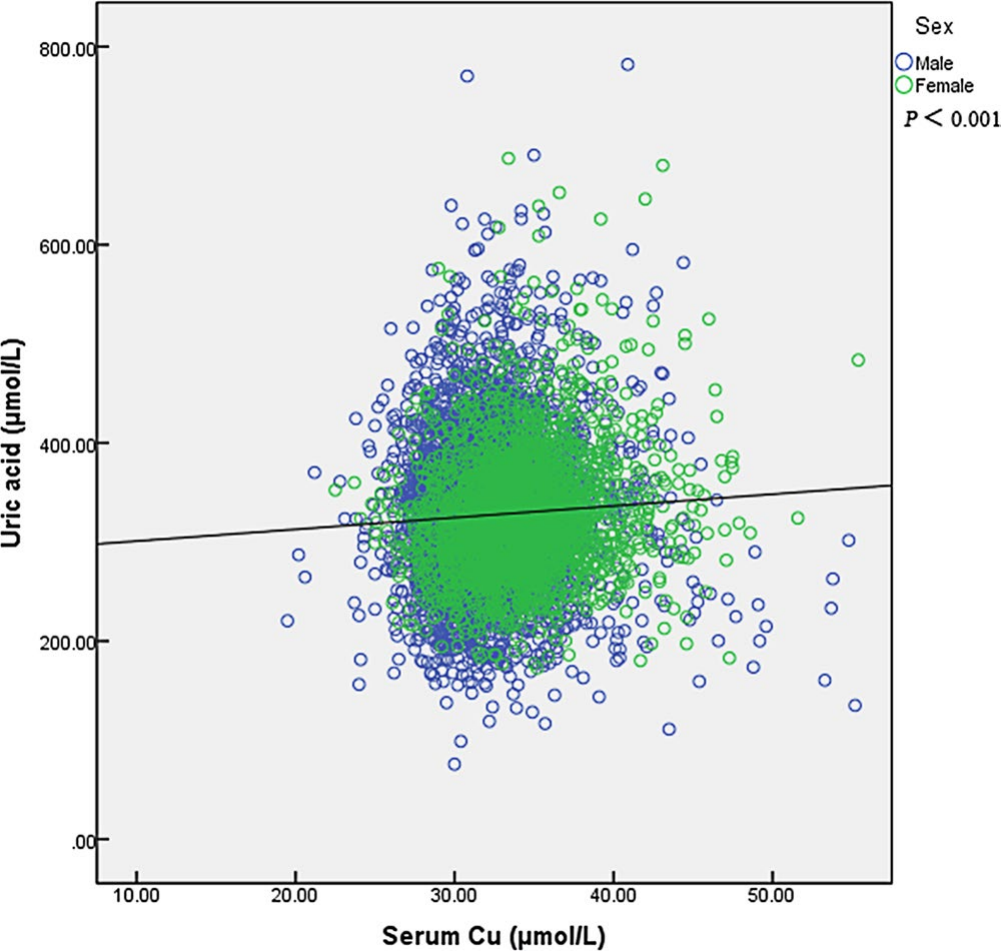
Scatter plot showing results of multivariable analyses, in which higher serum UA was associated with higher serum Cu. *P* values were obtained after adjustment for age, sex, BMI smoking, drinking, education, activity level, occupation, hypertension, diabetes. Cu, copper.

The outcomes of multivariable adjusted connections between serum Cu concentration and the prevalence of hyperuricemia are listed in Table 3. Comparing with the lowest quintile, age- and gender- adjusted ORs (Model 1) of the total population demonstrated significant increased prevalence of hyperuricemia in the third [OR 1.38 (95% CI: 1.12 to 1.70)], fourth [OR 1.37(95% CI: 1.07 to 1.66)] and highest [OR 1.53 (95% CI: 1.23 to 1.91)] quintile of serum Cu, with *P* for trend <0.001; significant increased prevalence of hyperuricemia was also observed in the third [OR 1.36 (95% CI: 1.10 to 1.68)], fourth [OR 1.34 (95% CI: 1.07 to 1.67)] and highest [OR 1.55, 95% CI: 1.24 to 1.94)] quintiles after adjustment for multiple confounders (Model 2) including age, sex, BMI, smoking, drinking status, with P for trend <0.001; by adding education, activity level, occupation, hypertension and diabetes, multivariable adjusted ORs (Model 3) exhibited similar results, significant higher risk of hyperuricemia was found in the third [OR 1.31 (95% CI: 1.06 to 1.62)], fourth [OR 1.30 (95% CI: 1.04 to 1.62)] and highest [OR 1.52 (95% CI: 1.21 to 1.90)] quintiles, with P for trend <0.001. Similar results were found in the male population, all three regression models revealed that men in the third, fourth and highest quintiles presented a higher risk of hyperuricemia than those in the lowest quartile. Compared with men in the lowest quartile of serum Cu, those in highest quartile had higher odds of prevalence of hyperuricemia [Model 1: OR 1.33 (95% CI: 1.03 to 1.72); Model 2: OR 1.35(95% CI: 1.04 to 1.76); Model 3: OR 1.35(95% CI: 1.04 to 1.77)], the *P* values for trend in Model 1 to 3 were 0.005, 0.004, 0.005, respectively. For the female population, compared with the lowest quintile, only the highest quintile showed significant increased prevalence of hyperuricemia [Model 1: OR 2.00 (95% CI: 1.16 to 3.43); Model 2: OR 1.94 (95% CI: 1.12 to 3.35); Model 3: OR 1.87 (95% CI: 1.08 to 3.23)], the *P* values for trend in Model 1 to 3 were 0.002, 0.003, 0.006, respectively. Results from multivariable models confirmed the positive association when serum Cu was considered as a continuous exposure variable (per 10 μmol/L increment, Table 3). In addition, serum Cu was significantly associated with UA (P < 0.001) after adjustment for age, sex, BMI, smoking, drinking, education, activity, occupation, hypertension and diabetes (Fig. 1).

**Table 3:** Multivariable-adjusted relationship between serum Cu and hyperuricemia.

	Quintiles of serum Cu					*P* for trend	Serum Cu, per 10 μmol/L increase
	Q1 (lowest)	Q2	Q3	Q4	Q5 (highest)		
Median serum Cu (μmol/L)	12.40	14.60	16.20	17.90	20.90	—	—
**Total**							
Model 1 (95% CI)	1.00 (reference)	1.16 (0.94, 1.43)	1.38 (1.12, 1.70)	1.34 (1.07, 1.66)	1.53 (1.23, 1.91)	<0.001	1.45 (1.20, 1.76), P < 0.001
Model 2 (95% CI)	1.00 (reference)	1.15 (0.93, 1.42)	1.36 (1.10, 1.68)	1.34 (1.07, 1.67)	1.55 (1.24, 1.94)	<0.001	1.50 (1.23, 1.81), P < 0.001
Model 3 (95% CI)	1.00 (reference)	1.11 (0.90, 1.38)	1.31 (1.06, 1.63)	1.30 (1.04, 1.62)	1.52 (1.21, 1.90)	<0.001	1.50 (1.23, 1.83), P < 0.001
**Male**							
Model 1 (95% CI)	1.00 (reference)	1.15 (0.93, 1.44)	1.49 (1.19, 1.86)	1.38 (1.08, 1.76)	1.33 (1.03, 1.72)	0.005	1.31 (1.05, 1.64), P = 0.017
Model 2 (95% CI)	1.00 (reference)	1.13 (0.90, 1.42)	1.44 (1.15, 1.81)	1.36 (1.07, 1.75)	1.35 (1.04, 1.76)	0.004	1.35 (1.07, 1.69), P = 0.010
Model 3 (95% CI)	1.00 (reference)	1.09 (0.87, 1.37)	1.39 (1.11, 1.75)	1.33 (1.04, 1.71)	1.35 (1.04, 1.77)	0.005	1.38 (1.10, 1.75), P = 0.006
**Female**							
Model 1 (95% CI)	1.00 (reference)	1.35 (0.73, 2.51)	1.12 (0.61, 2.06)	1.37 (0.77, 2.43)	2.00 (1.16, 3.43)	0.002	2.00 (1.38, 2.90), P < 0.001
Model 2 (95% CI)	1.00 (reference)	1.34 (0.72, 2.50)	1.11 (0.60, 2.05)	1.36 (0.76, 2.42)	1.94 (1.12, 3.35)	0.003	1.95 (1.34, 2.84), P < 0.001
Model 3 (95% CI)	1.00 (reference)	1.31 (0.70, 2.46)	1.13 (0.61, 2.09)	1.30 (0.73, 2.33)	1.87 (1.08, 3.23)	0.006	1.87 (1.28, 2.74), P = 0.001

OR, Odds ratio; CI, confidence interval; Cu, copper

Values are adjusted OR (95% CI) unless otherwise indicated.

Model 1 included age (continuous data), sex (male, female);

Model 2 included age (continuous data), sex (male, female), BMI (≥28 kg/m^2^, <28 kg/m^2^), smoking status (yes/no), and drinking status (yes/no) (age, BMI, smoking status and drinking status for the sex subgroup);

Model 3 added education level (with or above high school background or not), activity level (continuous data), occupation (manual worker/non-manual worker), hypertension (yes/no), diabetes (yes/no), on the basis of model.

## Discussion

The present cross-sectional study showed a positive association between serum Cu concentrations and the prevalence of hyperuricemia, and the findings remained consistent after adjustment for confounders.

Few studies have examined the association between Cu and serum UA, and the findings are still inconclusive. By conducted a population-based study with 1197 subjects aged between 45 to 64 years, Bo *et al*.^17^ noted that UA decreased significantly from the lowest to the highest tertile of Cu intake; however, in a randomly identified subgroup of male subjects (n = 231) from this study, Bo *et al*. did not find any significant association between serum Cu and serum UA. Unlike Bo *et al*., the present study indicated the prevalence of hyperuricemia was positively associated with serum Cu concentration, which may be attribute to our large sample size. Fields *et al*.^19^ found that rats with Cu-deficient diet exhibited high levels of plasma UA; while Derouiche *et al*.^20^ reported that Cu supplementation caused an augmentation of UA in rats with or without diabetes. However, the above animal studies only provided evidence for the relationship between dietary Cu and serum UA.

Under certain conditions, Cu plays its role as an activator of Xanthine oxidase (XO). XO is a key enzyme in purine metabolism, which can oxidize hypoxanthine from nucleic acid metabolites into xanthine, and xanthine into UA. Though several studies have documented the inhibition effect of Cu on XO^21–23^, in the later study conducted by Hadizadeh *et al*.^24^, Cu was proved to be both a reversible inhibitor and an activator of XO, a progressive reduction of catalytic efficiency was observed when the Cu^2+^ concentration raised from 5 to 700 μM.

The biological mechanisms linking serum Cu to hyperuricemia are unclear. It has been reported that UA may function either as an anti-oxidant (mainly in plasma) or pro-oxidant (mainly in cells)^25^. A high UA concentration may contribute to the antioxidant behavior of UA^16,26^, while both UA and hyperuricemia related pathologies, such as cardiovascular disease, hypertension and obesity, are associated with oxidative stress^8,9,12,27–29^. Therefore, UA may play a very complex role through the anti-oxidant and pro-oxidant effects in hyperuricemia. Cu^2+^ is able to oxidize UA directly^15^, Cu^2+^/LDL ratio may be an influence factor on pro- and anti-oxidant behaviors of UA^30^. The Cu-reduction ability of UA explains not only its pro-oxidant behavior but also, in part, its anti-oxidant activity^16^. Moreover, UA is a potent mediator of inflammation. A high concentration of UA has been demonstrated to mediate inflammation by stimulating inflammatory cytokines^31,32^ and the nuclear factor kappa B (NF-κB) pathway^33,34^. On the other hand, serum Cu level was identified to be positively correlated with inflammation activity markers^17,35,36^, and Cu was proved to activate NF-κB^37^ and enhance the production of pro-inflammatory cytokines^38–40^. Based on the analysis above, the serum Cu may promote the effect of UA through the inflammatory mechanism.

The present study is characterized with several strengths. First, it is the first research work performed on a large sample (6,212 subjects) that directly relates serum Cu to hyperuricemia. The findings of this study may provide new insights into the mechanism and treatment of hyperuricemia. Second, adjustments for some potential confounding factors, such as education, activity level, occupation, hypertension and diabetes, increased reliability of the results. It should be noted that our study had several potential limitations. First, based on the cross-sectional data provided in this study, we cannot draw a conclusion that reflect the causal correlations. Further intervention trials and prospective longitudinal studies are therefore expected to establish a causal relationship between serum Cu and hyperuricemia. Second, the total serum Cu concentration was detected in this study. While approximately 90–95% of the total amount of Cu in blood serum is strongly protein-bound, mostly with α2–globulin (ceruloplasmin)^13^, the rest of Cu remains non-bounded (non-ceruloplasmin Cu). Ogihara *et al*.^41^ observed an elevation of non-ceruloplasmin as well as a reduction of UA in three of four untreated patients with Wilson’s disease, but the sample size was too small (only 4 patients) and all subjects were in a Wilson’s disease condition. A further study focusing on the relationship between ceruloplasmin and non-ceruloplasmin Cu with serum UA is therefore suggested.

## Conclusion

In this population-based cross-sectional study, serum Cu concentration was positively associated with the prevalence of hyperuricemia. Further studies are required to estimate the exact mechanisms of the association between Cu and hyperuricemia pathogenesis.

## Data Availability

The data that support the findings of this study are available from the corresponding authors, TY and YW, upon reasonable request.
